# Self-Assembled 3D Flower-Like Nickel Hydroxide Nanostructures and Their Supercapacitor Applications

**DOI:** 10.1038/srep27318

**Published:** 2016-06-02

**Authors:** Nazish Parveen, Moo Hwan Cho

**Affiliations:** 1 School of Chemical Engineering, Yeungnam University, Gyeongsan, Gyeongbuk 712-749, South Korea

## Abstract

Three-dimensional (3D) nanostructures have attracted considerable attention because of their high surface areas and unique properties which gives outstanding performance in catalysis and energy storage applications. This paper proposes the growth mechanism of 3D flower-like β-Ni(OH)_2_ constructed through a two dimensional sheet framework using a one-step oleylamine-assisted solvothermal approach, where oleylamine acts as the surfactant, co-solvent, stabilizer, and reducing agent. A detailed examination of the product morphology after various reaction times suggested that the self-assembly of flower occurs through a mechanism involving nucleation, Ostwald ripening, and recrystallization. The associated characterization revealed it to be pure β-Ni(OH)_2_ without any sign of contamination. The effect of the morphology (sheet to 3D flower-like β-Ni(OH)_2_) on the electrochemical supercapacitive behavior was assessed by cyclic voltammetry and galvanostatic charge-discharge tests. The results showed that 3D flower-like β-Ni(OH)_2_ exhibited better specific capacitance of ~1567 F g^−1^ at a current density of 1 A g^−1^ and retained ~25% capacitance at a high current density of 10 A g^−1^ compared to the other reference materials. The superior electrochemical properties of the 3D flower-like β-Ni(OH)_2_ originate from their large specific surface area and unique structure.

Supercapacitors or electrochemical capacitors have attracted considerable attention for novel energy storage devices because they can immediately provide a higher power density with simultaneously shorter charging times than batteries, and a higher energy density than conventional dielectric capacitors^1^,^2^,^3^,^4^. Therefore, supercapacitors are considered promising energy storage devices and power supplies for digital products, hybrid electric vehicles, and other portable electronic devices that require a high power density and long cycle life^1^,^2^,^3^. In general, supercapacitors can be divided into the following two types according to their energy storage mechanism: electrical double layer capacitors (EDLC) typically made from carbon or graphene based materials, which work on rapid ion adsorption/desorption; and pseudocapacitors originating from fast faradaic charge transfer reactions similar to the processes observed in batteries^2^,^3^. Pseudocapacitors using transition-metal oxides and electronically conducting polymers as active materials can be used to produce asymmetrical supercapacitors with both improved energy and power densities^5^,^6^.

Earlier in supercapacitor applications, a range of metal oxides were used as electrode materials. Among them ruthenium oxide (RuO) is used widely as an electrode material because of its high theoretical capacitance (1500 Fg^−1^)^6^. On the other hand, the high cost and toxicity of RuO have limited its commercialization as a supercapacitive electrode material. Therefore, many studies have searched for alternative inexpensive electrode materials with good capacitive features, such as NiO, CoO_*x*_, Mn_3_O_4_, MnO_2_, CuO, Co(OH)_2_, and Ni(OH)_2_^7^,^8^,^9^. Among these metal oxides, Ni(OH)_2_ has attracted more attention as an electrode material in energy and power storage devices, particularly for supercapacitors, because of its unique physical and chemical properties, such as natural abundance, high theoretical surface area, low cost, and well-defined electrochemical redox behavior^7^,^10^,^11^. On the other hand, most Ni(OH)_2_-based electrodes have suffered from an inferior rate performance and poor cycle stability by possessing extremely attractive theoretical capacitance values. The utilization rate of the active materials and the electron and ion transmission rates determine the specific capacitance and rate performance of the supercapacitors, respectively. Therefore, the development and design of three dimensional structured materials have attracted considerable interest because they provide sufficient space and active sites for the interaction of electrolytes ions during the electrochemical process^12^,^13^. In addition, self-assembled micro/nano three-dimensional (3D) structures as electrode materials are some of the best systems in the area of supercapacitors^13^,^14^. These structures constructed with nanometer-scaled building blocks, including nanoparticles, nanorods, nanosheets etc., have the advantages of both the building blocks and assemblies. More importantly, the building blocks with a high specific surface area can guarantee more effective contact between the electrolyte and the active materials, hence increasing their utilization rate. The assembly, with its desirable and mechanical properties, guarantees good stability and practical fabrication^15^,^16^,^17^. Therefore, the development of a feasible and facile approach to manufacture morphology-controlled 3D flower-like β-Ni(OH)_2_ structures consisting of a low-dimensional building block is desired. Over the past few years, a range of approaches have been developed to synthesize micro/nano-structures with complex morphologies. Of these, the hydrothermal or solvothermal method is considered to be efficient because the shape and size of materials is easy to control^18^,^19^.

This paper reports a simple, cost effective, one-step oleylamine-assisted solvothermal approach for the synthesis of 3D flower-like β-Ni(OH)_2_, where oleylamine acts as the surfactant, co-solvent, stabilizer, and reducing agent. The effects of the reaction time on the resulting morphology have been studied systematically to rationalize the structural configuration. The effects of morphology on the electrochemical performance were also examined. The results showed that the 3D flower-like β-Ni(OH)_2_ electrode exhibited a high specific capacitance of 1567 F g^−1^ at a current density of 1 A g^−1^ and retained approximately 25% at a higher current density. The excellent capacitance of the 3D flower-like β-Ni(OH)_2_ was attributed to a 3D flower-like structure, which provides a high surface area and shorter conduction length for electrolytic ions.

## Results and Discussion

### Morphological Analysis

**Figure 1:**
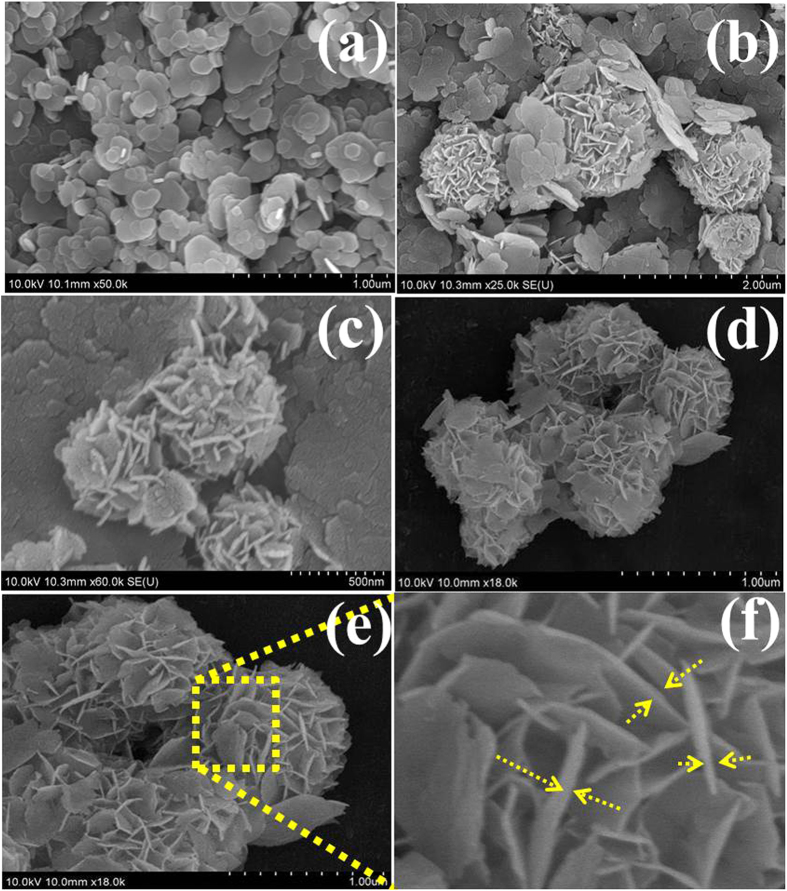
SEM images of the synthesized β-Ni(OH)_2_ for different reaction times: after 4 h (**a**), 8 h (**b,c**) and (**d–f**) 12 h having fully grown flowers fabricated and packed nanopetals.

Time-dependent experiments were conducted to understand the morphological changes and formation process of the 3D flower-like β-Ni(OH)_2_. During the experiment the samples were collected at different time intervals and the morphological changes was carried out by scanning electron microscope (SEM) and transmission electron microscopy (TEM) analysis. The initial morphological investigation of the as-synthesized 3D flower-like β-Ni(OH)_2_ for different reaction times was examined by SEM, which shows that the reaction time has a significant impact on the morphology of β-Ni(OH)_2_. As shown in Figs 1a and S1a, at the early stage after 4 h, the sample showed a sheet like structure with the size of ~200 to ~600 nm with no indication of the formation of flower-like structures. As the reaction time was increased from 4 h to 8 h (Figs 1b,c and S1b), several sheets began to form a flower-like morphology and the sheets acted as petals sticking out from the central part of the flower, and fewer sheets or ‘petals’ engaged in the building of a flower. After a 12 h reaction (Figs 1d–f and S1c), the growth of the 3D structures appeared to be complete, and these were now filled with packed sheets or ‘petals’. The colonial growth of these sheet or ‘petals’ produces flowers with compact constructions. These sheets or ‘petals’ are interconnected with each other and interlaced like petals on a flower. The surface of each petal is very smooth, probably due to Ostwald ripening. As described elsewhere, such nanosheet-based ordered 3D flowered structures allow high access of the electrolyte to the integrated nanosheets, which are essential for the decrease in interface contact resistance between the parallel directions of the electrodes and electrolyte^20^. An enlarged SEM image (Fig. 1f) of the Ni(OH)_2_ synthesized for a reaction time of 12h clearly shows that the 3D flower-like morphology generates some porosity. This SEM image shows that structures are comprised favorably of densely packed and uniform sheets with a thickness of less than 100 nm. The entire evaluation processes illustrate the growth of Ni(OH)_2_ to a 3D structure involving nucleation/growth^18^,^19^, aggregation mechanisms^20^,^21^, self-assembly, and Ostwald ripening^18^,^19^,^22^.

**Figure 2:**
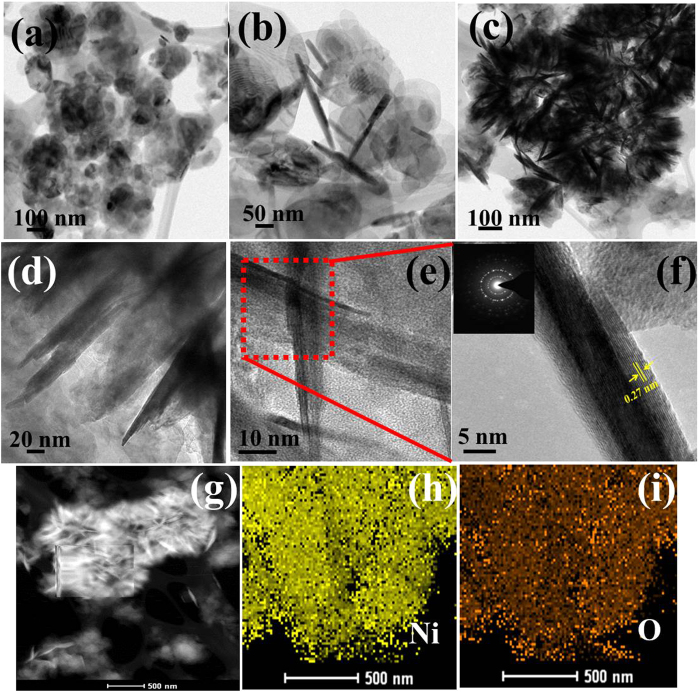
TEM images of the synthesized β-Ni(OH)_2_ at different reaction times: after 4 h (**a**), 8 h (**b**), 12 h having grown flower-like structures fabricated with packed nanopetals (**c**), segment of a 3D flower (**d**), HRTEM image of intersecting nanopetals (**e**), enlarged view of the enclosed area (SAED pattern in inset) (**f**) and elemental mapping (**g–i**) of the 3D flowers after 12 h.

Further TEM analysis was also performed to understand the construction of 3D flower-like β-Ni(OH)_2_, texture, and crystalline behavior of β-Ni(OH)_2_-4, β-Ni(OH)_2_-8, and 3D flower-like β-Ni(OH)_2_. Figure 2 shows TEM images of the β-Ni(OH)_2_ after different solvothermal treatments, i.e., 4, 8, and 12 h. After 4 h (Figs 2a and S2a) sheet-like structures were observed with no indication of flower formation but after 8 h (Figs 2b and S2b–d), both ‘petal’ and sheet-like structures were observed, which indicates that the sheet began to convert to a petal-like structure. In addition, after 12 h (Figs 2c and S2e), the complete formation of 3D flower-like structure was observed and these arrangements were made up of distinguishable sheets or ‘petals’ with a width ranging from 5 to 30 nm. The sharp contrast among the dark edges of these petals and their faded area clearly confirm that the some porous structures are generated in the 3D flowers, which substantiates the compactness of the petals preferentially in the central region of the flower rather than in the exterior, indicating the density variations of the sheet inside the same porous structure. These features associated with the interweaving petal subunits showed good agreement with the SEM observations. Figure 2e presents a typical higher magnification TEM image of a piece of extended petals of the flower-like structures. An enlarged view in Fig. 2f clearly shows that the petals are thin. A close inspection of Fig. 2e revealed the lattice-resolved HRTEM images of the regional part surrounded by a red frame. The insets clearly show the atomic planes with a fringe spacing of ~0.27 nm. The corresponding SAED results (inset in Fig. 2f) present a ring like diffraction pattern of Ni(OH)_2_ flowers, indicating the polycrystalline nature or structure. The chemical composition of these 3D flower-like β-Ni(OH)_2_ was determined by elemental mapping and energy dispersive X-ray (EDX) analysis, as shown in Figs 2g–i and S3. Elemental mapping suggested the presence of Ni and O in the 3D flower-like β-Ni(OH)_2_, and the EDX spectrum (Fig. S3) also showed only a Ni and O peak, which further confirmed that the 3D flower-like β-Ni(OH)_2_ had been synthesized successfully using one-pot solvothermal method and would potentially exhibit superior performance in supercapacitor applications.

### Growth mechanism for the formation of 3D flower-like β-Ni(OH)_2_ structure

SEM and TEM indicated that the possible crystal formation and its growth of the β-Ni(OH)_2_ as a 3D flower-like β-Ni(OH)_2_ occurred in two stages. First, Ni(OH)_2_ with a poor crystalline phase was developed via the initial reaction of all the precursor, i.e., Ni(NO_3_)_2_.6H_2_O, ethanol, and oleylamine. Oleylamine acts as a surfactant, solvent, and reducing agent, and forms complex compounds with the metal ions of the metal precursor, leading to metastable compounds that can act as secondary precursors.

**Figure 3:**
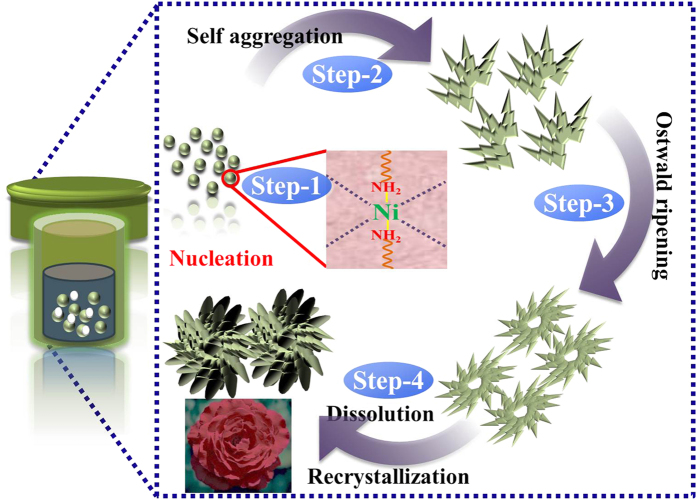
Schematic diagram of stepwise growth mechanism and morphological evolution of a 3D flower-like β-Ni(OH)_2_ structure.

The 3D flower-like morphology of the Ni(OH)_2_ was attained simply by the changing the reaction duration i.e. from 4 h to 12 h. The Ni(OH)_2_ obtained from the 4 h reaction time only showed a two-dimensional sheet-like morphology with a sheet size of 400 to 600 nm (Fig. 1a). When the reaction time was extended from 4 h to 8 h, several two-dimensional sheets began to convert to the flower morphology, as depicted in Fig. 1b. When the reaction time was increased to 12 h, the thickness of the interconnected was increased and the pore size of the surrounded petals decreased concomitantly, which led to the formation of compact three-dimensional flower structures (Fig. 1c). When the reaction time was prolonged, the size of the 3D structures increased and the morphology became a flower-like structure with a network of sheets on their surfaces, whereas after 12 h, the flower-like morphology gradually became more prominent^18^. These morphological changes from sheet to 3D flower-like structures indicate that the mechanism includes nucleation/growth^18^,^19^, aggregation^20^,^21^, self-assembly, and Ostwald ripening^18^,^19^,^22^. Among these, the Ostwald ripening and self-assembly played important roles to form the 3D flower-like structure of the Ni(OH)_2_ from its the initial crystal structure. In the Ostwald ripening process, flakes with larger size and small surface energy are thermodynamically favored and the dissolved tiny and unstable crystals provides the source material for the growth of flakes during the dissolution and recrystallization process. The dissolved nickel atoms may continuously attach and bond to the surface of larger flakes, and spread the (001) planes in order to achieve a minimum total free energy^23^. Overall, the larger flakes with low surface energy are thermodynamically favored and the unstable tiny crystals dissolved to provide the source material for the growth of flakes during the dissolution and recrystallization process. The dissolved nickel atoms may be able to attach continuously and bond to the surface of the larger flakes, and spread the (001) planes to reduce the total free energy^23^. A similar mechanism has also been reported for the synthesis of 3D structures that involve rapid nucleation followed by the slow aggregation and crystallization of the initial crystal structure^24^. Figure 3 presents the proposed growth mechanism of the 3D flower-like β-Ni(OH)_2_.

### Structural Analysis

**Figure 4:**
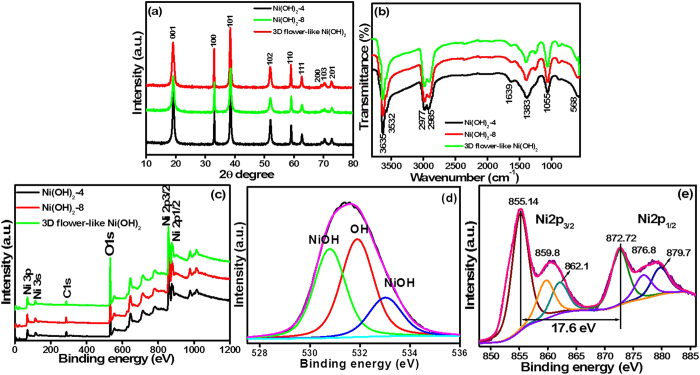
(**a**) XRD pattern, (**b**) FTIR analysis, (**c**) XPS survey spectra of the β-Ni(OH)_2_-4, β-Ni(OH)_2_-8 and 3D flower-like β-Ni(OH)_2_, (**d**) O 1s & (**e**) Ni 2p core level spectra of 3D flower-like β-Ni(OH)_2_.

Figure 4a presents the XRD patterns of the as-synthesized β-Ni(OH)_2_ prepared by the solvothermal method for different reaction times (4, 8 & 12 h) at 180 °C. β-Ni(OH)_2_-4, β-Ni(OH)_2_-8, and 3D flower-like β-Ni(OH)_2_ showed XRD peaks at ~19.15, 33.02, 38.49, 52.03, 58.94, 62.63, 69.27, 70.45, and 72.81° 2θ. The peak became more intense with increasing reaction time (4, 8 & 12 h). For 3D flower-like β-Ni(OH)_2_, peaks at 19.15, 33.02, 38.49, 52.03, 58.94, 62.63, 69.27, 70.45, and 72.81° 2θ were assigned to the (001), (100), (101), (102), (110), (111), (200), (103) and (201) planes, respectively, which were in good agreement with the standard values (JCPDS file no. 14-0117). No obvious peak from other impurities, such as α-Ni(OH)_2_, was observed, indicating the high purity of the synthesized product^25^. 3D flower-like β-Ni(OH)_2_ could be indexed entirely to a C6-type structure with the crystal phase of β-Ni(OH)_2_ having unit cell dimensions of a_0_ = 3.126 Å and c_0_ = 4.605 Å^24^,^26^,^27^. In addition, the (101) XRD peak for 3D flower-like β-Ni(OH)_2_ was more intense compared to the (001) plane. This suggests that the sheets are grown preferentially along the (001) plane by Ostwald ripening. These XRD results are consistent with the previously reported articles^23^.

FTIR spectroscopy was used to probe the major functional groups and the chemical interaction of the samples. Figure 4b shows the FTIR spectra of all the β-Ni(OH)_2_ samples, in which the sharp band observed at ~3635 cm^−1^ was assigned to the stretching vibrational mode (ν-OH) of the non-hydrogen bonded hydroxyl groups, whereas the broad band at ~3495 cm^−1^ was attributed to the stretching mode of the hydrogen bonded hydroxyl groups in the Ni(OH)_2_ structure. The peak at ~550 cm^−1^ was attributed to the in plane deformation vibrations of water (δ-OH). Weak bands were observed at ~2885 and ~2977 cm^−1^, which were assigned to the symmetric and asymmetric stretching vibrations of the -CH_2_- alkyl chain. The presence of these -CH_2_- alkyl chain may be due to the use of oleylamine in the reaction as a surfactant and reducing agent^28^.

Nitrogen adsorption-desorption isotherms were conducted to examine the structural characteristics and surface area of the β-Ni(OH)_2_-4, β-Ni(OH)_2_-8, and 3D flower-like β-Ni(OH)_2_ in Figure S4. The BET surface area of the β-Ni(OH)_2_-4, β-Ni(OH)_2_-8, and 3D flower-like β-Ni(OH)_2_ were ~38.9 m^2^/g, ~40.8 m^2^/g, and ~72.9 m^2^/g, respectively. This confirms that the 3D flower-like β-Ni(OH)_2_ possess a relative large specific surface area due to the well-defined interior spaces caused by the unique 3D flower-like ordered configuration, which is beneficial to the electrochemical property. Furthermore, the BET analysis clearly shows that the 3D flower β-Ni(OH)_2_ has a two-fold higher surface area than β-Ni(OH)_2_-4. Overall, the special 3D flower-like structure will provide sufficient space and network-like petals for the interaction and intercalation of the electrolytic ions during the electrochemical performance, which would be advantageous for enhancing the supercapacitive performance.

XPS was performed to determine the surface characteristics, chemical composition, i.e., types of carbon, oxygen and nickel bonds, as well as the percentage of oxygen and nickel present in the synthesized Ni(OH)_2_-4, β-Ni(OH)_2_-8, and 3D flower-like β-Ni(OH)_2_. The XPS survey scans (Fig. 4c) provide a complete view of the surface elemental composition of the β-Ni(OH)_2_ samples (4 h, 8 h, and 12 h); no impurities were observed on the Ni(OH)_2_ surface. As shown in Fig. 4c, the survey spectra of Ni(OH)_2_-4, β-Ni(OH)_2_-8, and 3D flower-like β-Ni(OH)_2_ confirm the presence of only Ni and O, which is consistent with the EDX and elemental mapping results. The high-resolution O 1s (Fig. 4d) and Ni 2p (Fig. 4e) fitted core-level spectra of the 3D flower-like β-Ni(OH)_2_, and characteristic peaks of Ni 2p and O 1s spectra were assigned to Ni^2+^ and hydroxyl ions^29^,^30^.

Figure 4d presents the fitted O 1s core level spectra of the 3D flower-like β-Ni(OH)_2_, which shows the three distinct peaks at a binding energy of 530.76, 531.88, and 533.01 eV. The binding energy observed at 530.76 eV was assigned to the metal-bond in 3D flower-like β-Ni(OH)_2_, whereas the high intensity peak observed at a binding energy of 531.88 eV was assigned to the Ni-(OH) bond. Similarly, the peak at a binding energy of 533.01 eV was assigned to the multiplicity of the physisorbed and chemisorbed water at or near the surface of β-Ni(OH)_2_^29^,^30^,^31^,^32^. These results show that many adsorbed hydroxyl group are present on the Ni surface, which may help enhance the electrochemical performance of the materials.

The high resolution Ni 2p core-level spectrum (Fig. 4e) was obtained to provide more details of the nature of the chemical bonding and the types of nickel present on the β-Ni(OH)_2_. The core-level spectrum of the Ni 2p was split into two major peaks at ~872.7 and ~855.1 eV, which were assigned to Ni 2p_1/2_ and Ni 2p_3/2_, respectively, with a spin energy separation of 17.6 eV. Satellite peaks (Ni 2p_1/2_, satellite: ~876.8 and ~879.7 eV; Ni 2p_3/2_, satellite: ~859.8 and ~862.1 eV) were also observed, which are associated with the two types of nickel species and were assigned to Ni(II) and Ni(III) ions^32^.

The thermal behavior of the as-prepared 3D flower-like β-Ni(OH)_2_ was examined by TGA and DTA (Figure S5). The TGA curve of the 3D flower-like β-Ni(OH)_2_ showed two weight-loss regions. The first weight-loss region was observed at ~240 to ~300 °C, which was attributed to the desorption of physisorbed water on the Ni(OH)_2_ surface. The second weight-loss region was in the range, 300 to 480 °C. A large endothermic peak was observed at 300 °C for the sample in the DTA curves. The temperature range of the endothermic peak in the DTA curve fitted well with that of the weight loss in the TGA curve, corresponding to endothermic behavior during the decomposition of β-Ni(OH)_2_ to NiO via the following reaction^33^.


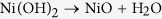


The weight of the sample decreased continuously up to ~600 °C, after which no obvious weight loss was observed. The total weight loss below 500 °C was 20.6%, which is slightly higher than the theoretical value of 19.5% calculated from the decomposition reaction of Ni(OH)_2_ to NiO, indicating that the complete decomposition of Ni(OH)_2_ was achieved at 600 °C^21^.

### Electrochemical performance

**Figure 5:**
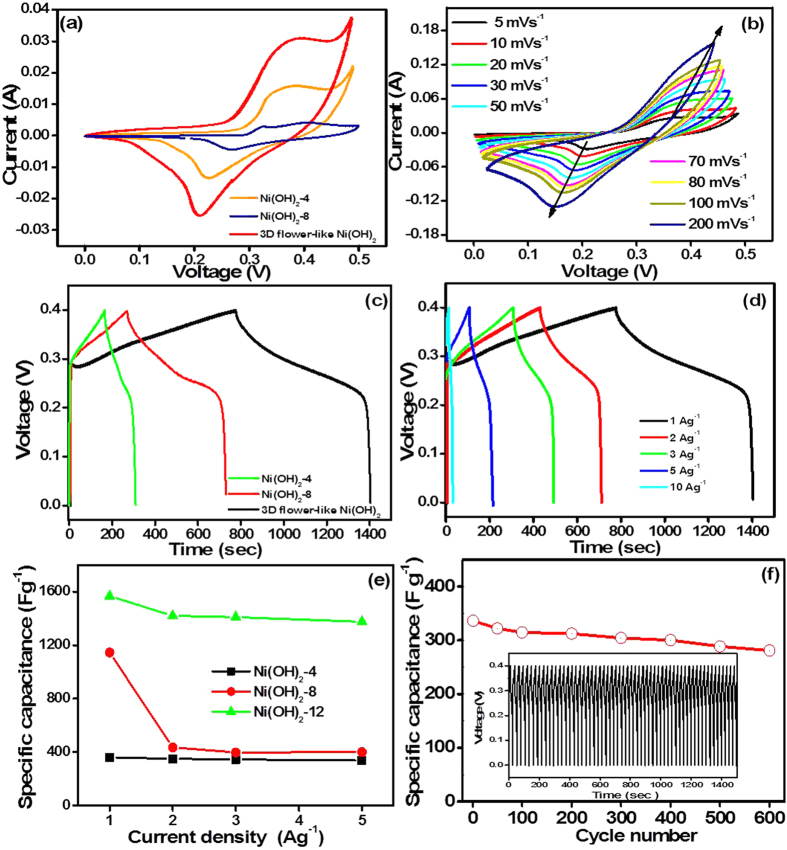
Electrochemical characterization of β-Ni(OH)_2_: (**a**) Cyclic voltammograms of Ni(OH)_2_-4, Ni(OH)_2_-8 and 3D flower-like Ni(OH)_2_ at a scan rate of 5 mV s^−1^ (**b**) 3D flower-like β-Ni(OH)_2_ at a scan rate of 5–200 mV s^−1^, (**c**) CD curves at a current density of 1 A g^−1^ (**d**) CD curves of 3D flower-like β-Ni(OH)_2_ at different current densities, (**e**) specific capacitance of Ni(OH)_2_-4, Ni(OH)_2_-8 and 3D flower-like β-Ni(OH)_2_, and (**f**) cyclic stability of 3D flower-like β-Ni(OH)_2_.

The morphological effects of Ni(OH)_2_-4, Ni(OH)_2_-8, and 3D flower-like β-Ni(OH)_2_ electrodes on the electrochemical capacitance were examined by CV and CD measurements, which is the most prominent tool for examining the capacitance property of the materials. CV is generally considered a suitable tool for determining the oxidation reduction behavior of the materials. Figure 5a presents the CV curves of the Ni(OH)_2_-4, Ni(OH)_2_-8, and 3D flower-like β-Ni(OH)_2_ electrodes, at a 5 mVs^−1^ scan rate over the potential range, 0.0 to 0.5V. Figure 5b shows the CV curves of the 3D flower-like β-Ni(OH)_2_ and Figure S6a,b shows the CV curve of the Ni(OH)_2_-4 and Ni(OH)_2_-8 at various scan rates ranging from 5–200 mV s^−1^. All CV curves showed a pair of redox peaks, such as the anodic peak (positive current density) at approximately ~0.45 V, due to the oxidation of Ni(OH)_2_ to NiOOH, whereas the cathodic peak (negative current density) at approximately ~0.19 V was assigned to the reverse reduction process, suggesting that the specific capacitance characteristic is governed mainly by the Faradic process^34^,^35^,^36^. Among these prepared electrodes, the 3D flower-like β-Ni(OH)_2_ electrode possessed a significantly larger enclosed area than that of the β-Ni(OH)_2_-4 and β-Ni(OH)_2_-8 electrodes. This enhanced performance of the 3D flower-like β-Ni(OH)_2_ electrode might have two explanations: (i) the three dimensional structure of the 3D flower-like β-Ni(OH)_2_, which provides a larger surface area and better pore size than pure β-Ni(OH)_2_-4 and β-Ni(OH)_2_-8 (Figure S4), and can expose more active sites for the intercalation of ions; and (ii) the oriented crystallinity of 3D flower-like β-Ni(OH)_2_ with a homogeneous elemental distribution may also enhance those electron intercalation/deintercalation processes (Figs 2 and S2).

The specific capacitance was examined further by CD to highlight the capacitance of the β-Ni(OH)_2_-4, β-Ni(OH)_2_-8, and 3D flower-like β-Ni(OH)_2_ over the potential range, 0.0 to 0.4 V. Figure 5c presents the CD curve of β-Ni(OH)_2_-4, β-Ni(OH)_2_-8, and 3D flower-like β-Ni(OH)_2_ at a fixed current density at 1 A g^−1^. The 3D flower β-Ni(OH)_2_ showed much higher capacitance performance ~1567 F g^−1^ at a current density of 1 A g^−1^ than the β-Ni(OH)_2_-4, β-Ni(OH)_2_-8, which is ~360 F g^−1^ and 1145 F g^−1^, respectively, and might be due to its unique 3D flower-like structure. Furthermore, Fig. 5d shows the charge/discharge curves of the 3D flower-like β-Ni(OH)_2_, whereas Figure S7a,b shows β-Ni(OH)_2_-4 and β-Ni(OH)_2_-8 at different current densities from 1 to 10 A g^−1^. The specific capacitance of β-Ni(OH)_2_-4 at a current density of 1, 2, 3, 5, and 10A g^−1^ was found to be 360, 350, 345, 337, and 225 F g^−1^, respectively, whereas for β-Ni(OH)_2_-8, it was 1145, 435, 400, 397, and 275 F g^−1^, respectively. Similarly, the specific capacitance of the 3D flower-like β-Ni(OH)_2_ was ~1567, 1420, 1410, 1375, and 336 F g^−1^ at a current density of 1, 2, 3, 5, and 10 A g^−1^. In particular, the 3D flower β-Ni(OH)_2_ exhibited excellent capacitance performance because of its unique 3D structure, which provides a large surface area and better pore size that may offer more active sites for the intercalation of the electrolyte and can maximize its utilization as an electrode material. Furthermore, 3D flower-like β-Ni(OH)_2_ retained ~25% capacitance, even at a high current rate of 10 A g^−1^. Figure 5e presents the specific capacitance plotted as a function of the current density for β-Ni(OH)_2_. The specific capacitance decreased with increasing current density owing to the decreased the penetration of the electrolyte into the pores of the electrode materials. For the 3D flower-like β-Ni(OH)_2_, the apparent plateau region was observed at voltages greater than 0.4 V at a low current density of 1 A g^−1^ due to the oxidation of Ni(OH)_2_ to NiOOH (Fig. S8), which is consistent with the CV results^11^,^37^,^38^,^39^,^40^,^41^,^42^. Table 1 lists the difference in capacitance between the 3D flower-like β-Ni(OH)_2_ and other reported α-Ni(OH)_2_ and β-Ni(OH)_2_-based materials. Compared to previous reports, the 3D flower-like β-Ni(OH)_2_ exhibited better performance than most of the capacitors reported previously.

Figure 5f represents the specific capacitances of 3D flower-like β-Ni(OH)_2_ according to the cycling condition at a current density of 10 A g^−1^. The specific capacitance of 3D flower-like β-Ni(OH)_2_ (Fig. 5f) was as high as 90% after 600 cycles. This suggests that the self-assembled building blocks of 3D flower-like β-Ni(OH)_2_ may be more beneficial to enhancing the electrochemical performance than other morphologies, such as nanoparticles, nanoplates, nanospheres, etc. The excellent electrochemical performance of the as-prepared 3D flower-like β-Ni(OH)_2_ is due to their unique structure. The 3D structure may contribute to the improved electrochemical performance. The open space between the adjacent sheets allows for the easy diffusion of the electrolyte, which ensures that every sheet can participate in the electrochemical reaction because every sheet is in contact with the electrolyte. The 3D structure composed of the centrifugally self-assembled nanosheets could effectively increase the electrode/electrolyte contact area and facilitate the rapid transport of ions and electrons. The high specific surface area and self-assembled sheets structures during the charge-discharge process are beneficial for the better electrical conductivity and high structural stability, leading to the high specific capacitance, and good cycling performance of the 3D flower-like β-Ni(OH)_2_. The outstanding performance of the 3D flower-like β-Ni(OH)_2_ can be attributed due to the unique self-assembled sheet-like structures shortening the diffusion length of the electrons and ions, and the large-area material-electrolyte contact. Their comparatively large surfaces can also benefit the charge-transfer rate. Therefore, in addition to the intrinsic nature of the materials, the superior electrochemical properties of the 3D flower-like β-Ni(OH)_2_ originate from their large specific surface area and unique structure.

## Conclusions

A one-pot oleylamine-assisted solvothermal approach was used to fabricate 3D flower-like β-Ni(OH)_2_ assembled by a 2D sheet framework. The proposed methodology is a simple, single-step synthesis, and more importantly, oleylamine acts as both a reducing agent and surfactant in the synthesis process. The advantageous properties associated with the 3D flower-like structure are the larger surface area and better pore size, which can expose more active sites for the intercalation of ions, and the crystallinity of the 3D flower-like β-Ni(OH)_2_ with a homogeneous distribution of elements also enhances the intercalation/deintercalation process leading to outstanding electrochemical properties, such as high specific capacitance, capability, and charge/discharge stability of the 3D flower-like β-Ni(OH)_2_. The unique flower-like structures of β-Ni(OH)_2_ can be extended further to a wide range of applications, such as sensors, electronic devices, hydrogen storage, catalysis, and lithium-ion batteries.

## Experimental

### Materials

Nickel nitrate (Ni(NO_3_)_2_.6H_2_O), oleylamine (C_18_H_3_NH_2_), and cyclohexane (C_6_H_12_) were acquired from Sigma Aldrich, South Korea. Methyl alcohol and ethyl alcohol were supplied by Duksan Pure Chemicals Co. Ltd., South Korea and used as received. The de-ionized water used in these experiments was obtained from a PURE ROUP 30 water purification system.

### Methods

The changes in morphology from sheet to 3D flower-like β-Ni(OH)_2_ were observed by scanning electron microscopy (SEM, HITACHI-S4800) and the internal structure of β-Ni(OH)_2_ was examined by field emission transmission electron microscopy (FE-TEM, Tecnai G2 F20, FEI, USA). Phase analysis was performed by X-ray diffraction (XRD, PANalytical, X’Pert-PRO MPD, Netherland) using Cu Kα radiation (λ = 0.15405 nm). The functional groups and their interactions were examined by Fourier transform infrared (FTIR, Excalibur series FTS 3000 Bio-Rad spectrometer) spectroscopy. The textural properties were measured according to a N_2_ adsorption-desorption method using a volumetric gas adsorption apparatus (ASAP 2020, Micromeritics Inc. USA). The chemical state and surface composition were analyzed by X-ray photoelectron spectroscopy (XPS, ESCALAB 250 XPS system, Thermo Fisher Scientific U.K.) using monochromatized Al l Kα (hν = 1486.6 eV). Thermogravimetric analysis (TGA, Perkin Elmer, Pyris Diamond) was performed by heating the samples from 20 °C to 800 °C at a rate of 10 °C/min with an air flow rate of 200 mL min^−1^. The electrochemical properties were examined by cyclic voltammetry (CV) and charge/discharge (CD) using a potentiostat (Versa STAT 3, Princeton Research, USA).

### Experimental details for the synthesis of β-Ni(OH)_2_ nanostructures

The β-Ni(OH)_2_ nanostructures were synthesized by adapting the oleylamine-assisted hydrolysis of aqueous nickel nitrate with the assistance of a solvothermal method based on “bottom up” approach chemistry. In a typical preparation of β-Ni(OH)_2_, 1mM Ni(NO_3_)_2_.6H_2_O and 20 ml ethanol were placed in a beaker with magnetic stirring for 10 min. Subsequently, 2 ml of oleylamine and 15 ml of ethanol were added to form the precursor solution of the nickel oleylamine complex. The homogeneous solution was then transferred to a 100 mL Teflon-lined autoclave. The autoclave was sealed and maintained at 180 °C for different times (4 to 12 h) and cooled to room temperature. The green samples were collected and washed rapidly with cyclohexane, distilled water and ethanol to remove the organics, ions, and possible impurities. The samples were then dried at 80 °C for 6 h. The samples prepared at different times, such as after 4h, 8h, and 12h, were labeled β-Ni(OH)_2_-4, β-Ni(OH)_2_-8, and 3D flower-like β-Ni(OH)_2_, respectively

### Electrode preparation and electrochemical measurements

All electrochemical measurements were taken on a potentiostat Versa STAT 3, Princeton Research, USA. The electrochemical tests were carried out in a three-electrode cell with an aqueous 2M KOH solution as the electrolyte and a Pt plate and a saturated AgCl/Ag (3M KCl) as the counter and reference electrodes, respectively. The active electrodes (β-Ni(OH)_2_-4, β-Ni(OH)_2_-8, and 3D flower-like β-Ni(OH)_2_ were prepared by mixing the electroactive material (90 wt.%) and polytetrafluoroethene (10 wt.%) with isopropyl alcohol and pasted on nickel foam. Cyclic voltammetry (CV) was performed at scan rates of 5 mVs^−1^-200 mVs^−1^ over the potential range, 0 to 0.5V. The galvanostatic charge-discharge (CD) measurements were carried out at current densities ranging from 1 A g^−1^ to 10 A g^−1^ and a potential window of 0 to 0.4 V. The integrated-average gravimetric capacitance of each electrode was calculated from the charge discharge equation^2^.

## Additional Information

**How to cite this article**: Parveen, N. and Cho, M. H. Self-Assembled 3D Flower-Like Nickel Hydroxide Nanostructures and Their Supercapacitor Applications. *Sci. Rep.* **6**, 27318; doi: 10.1038/srep27318 (2016).

## Supplementary Material

Supplementary Information

## Figures and Tables

**Table 1:** Comparative capacitance of 3D flower-like β-Ni(OH)_2_ with other reported α and β-Ni(OH)_2_ structures.

S. no.	Structural morphology	Electrolyte	Specific Capacitance F g^−1^	Current density A g^−1^	Cyclic stability		Ref.
					Cycle Number	Retention %	
1	NiO	6M KOH	555	2	2000	–	11
2	Ni(OH)_2_ nanospheres	1M KOH	694.5	1	1000	90	37
3	Ni(OH)_2_ nanoplates	2M NaOH	793	1	–	–	38
4	Ni(OH)_2_ nanoparticles	2M KOH	291	0.5 mA cm^−2^	500	82	39
5	Hexagonal Na(OH)_2_	3M KOH	578	2.5 mA	400	95	40
6	β-Ni(OH)_2_ nanoparticles	1M KOH	715.3	0.5	–	–	41
7	Ni(OH)_2_ nanowires	6M KOH	833	5 mA cm^−2^	–	–	42
8	3D flower-like β-Ni(OH)_2_	2M KOH	1567	1	600	90	Present work
