# Supplementary material for: Self-Assembled 3D Flower-Like Nickel Hydroxide Nanostructures and Their Supercapacitor Applications
Source: Sci Rep. 2016 Jun 2;6:27318. doi: 10.1038/srep27318 (PMC4890008; doi:10.1038/srep27318)
Supplement: Supplementary Information [file srep27318-s1.pdf]

Supporting Information

Self-Assembled 3D Flower-Like Nickel Hydroxide Nanostructures and Their Supercapacitor Applications

Nazish Parveen and Moo Hwan Cho*

School of Chemical Engineering, Yeungnam University, Gyeongsan-si, Gyeongbuk 712-749, South Korea, Phone: +82-53-810-2517; Fax: +82-53- 810-4631.

*Corresponding author: mhcho@ynu.ac.kr

SEM image of β-Ni(OH)$_2$ at different reaction time duration

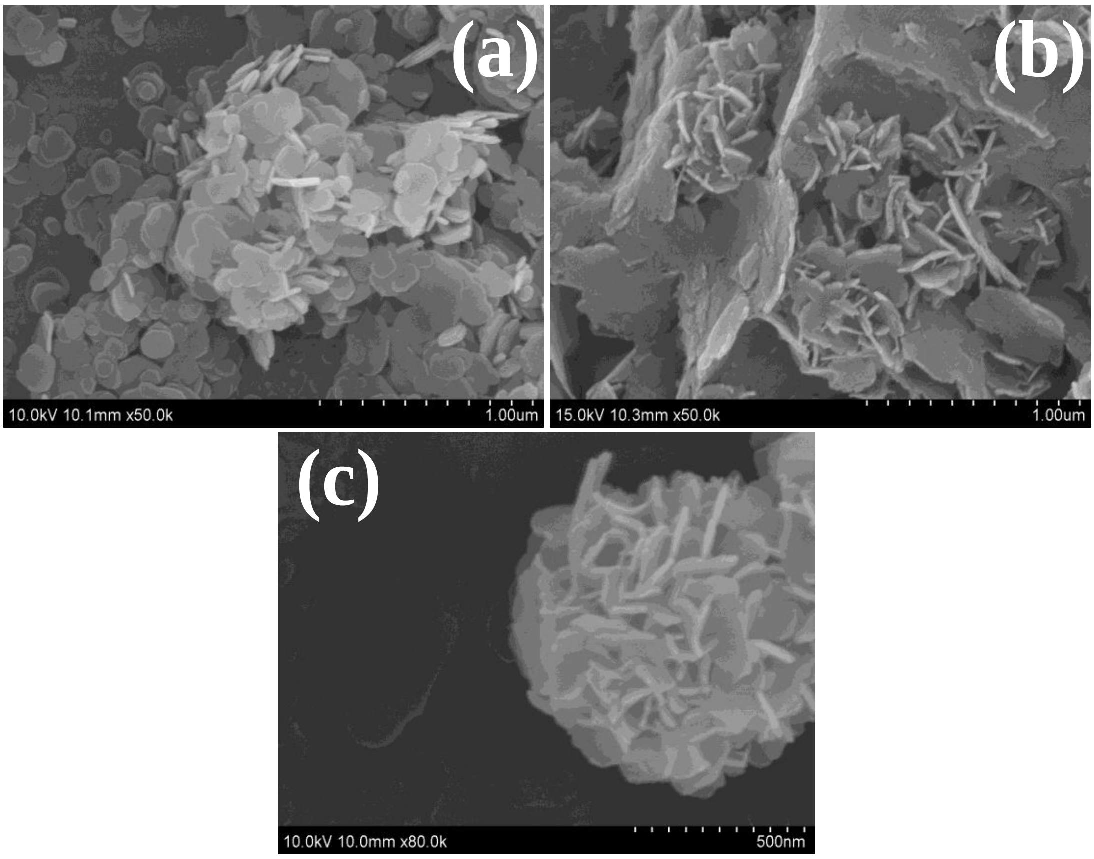

Figure S1. SEM image of β-Ni(OH)$_2$ at different reaction time duration: after (a) 4 h, (b) 8 h and (c) 12 h.

TEM image of β-Ni(OH)$_2$ at different reaction time duration

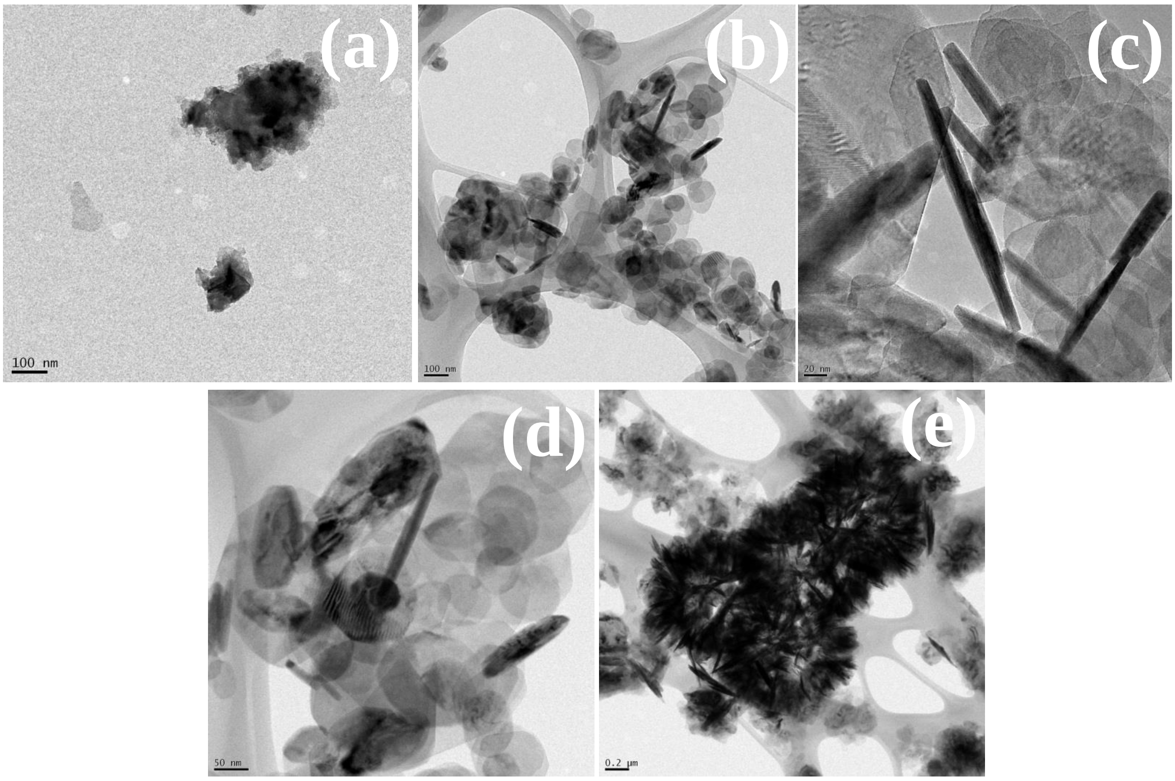

Figure S2. TEM image of β-Ni(OH)$_2$ at different reaction time duration: after (**a**) 4 h, (**b-d**) 8 h and (**e**) 12 h.

EDX of 3D flower like β-Ni(OH)$_2$-12

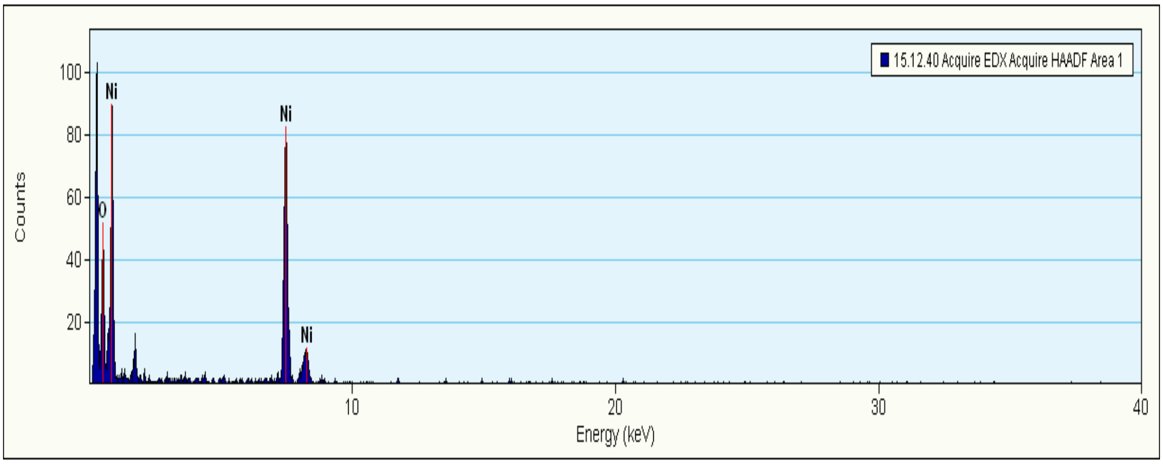

Figure S3. EDX of 3D flower like β-Ni(OH)$_2$-12

Nitrogen adsorption-desorption spectra

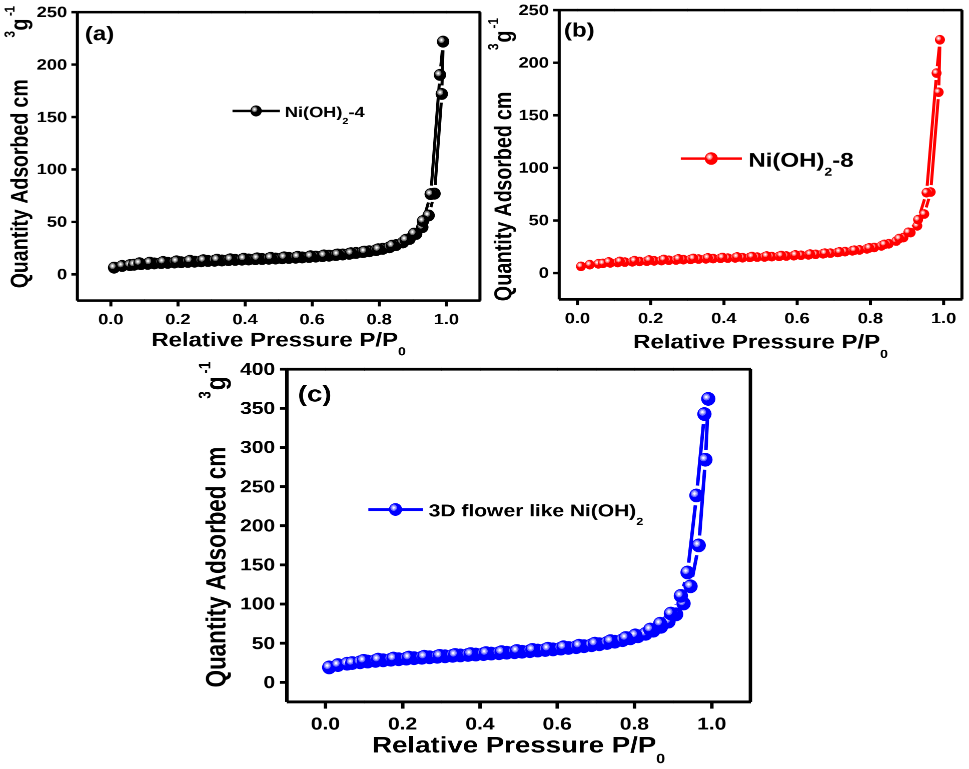

Figure S4. Nitrogen adsorption-desorption spectra of β-$Ni(OH)_2$-4, β-$Ni(OH)_2$-8 and 3D flower like β-$Ni(OH)_2$-12.

TGA and DTA analysis of 3D flower like β-$Ni(OH)_2$-12

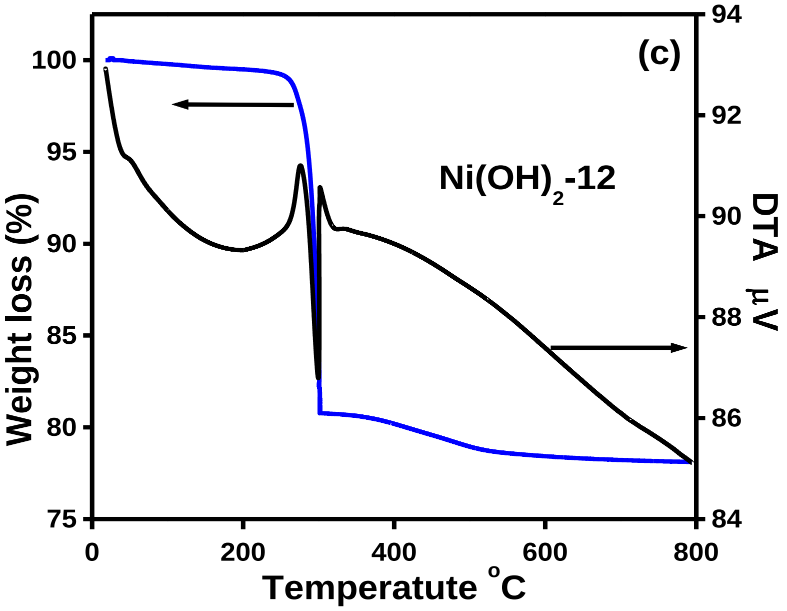

Figure S5. TGA and DTA of 3D flower like β-$Ni(OH)_2$-12.

Cyclic Voltammograms of β-Ni(OH)$_2$-4 and (b) β-Ni(OH)$_2$-8

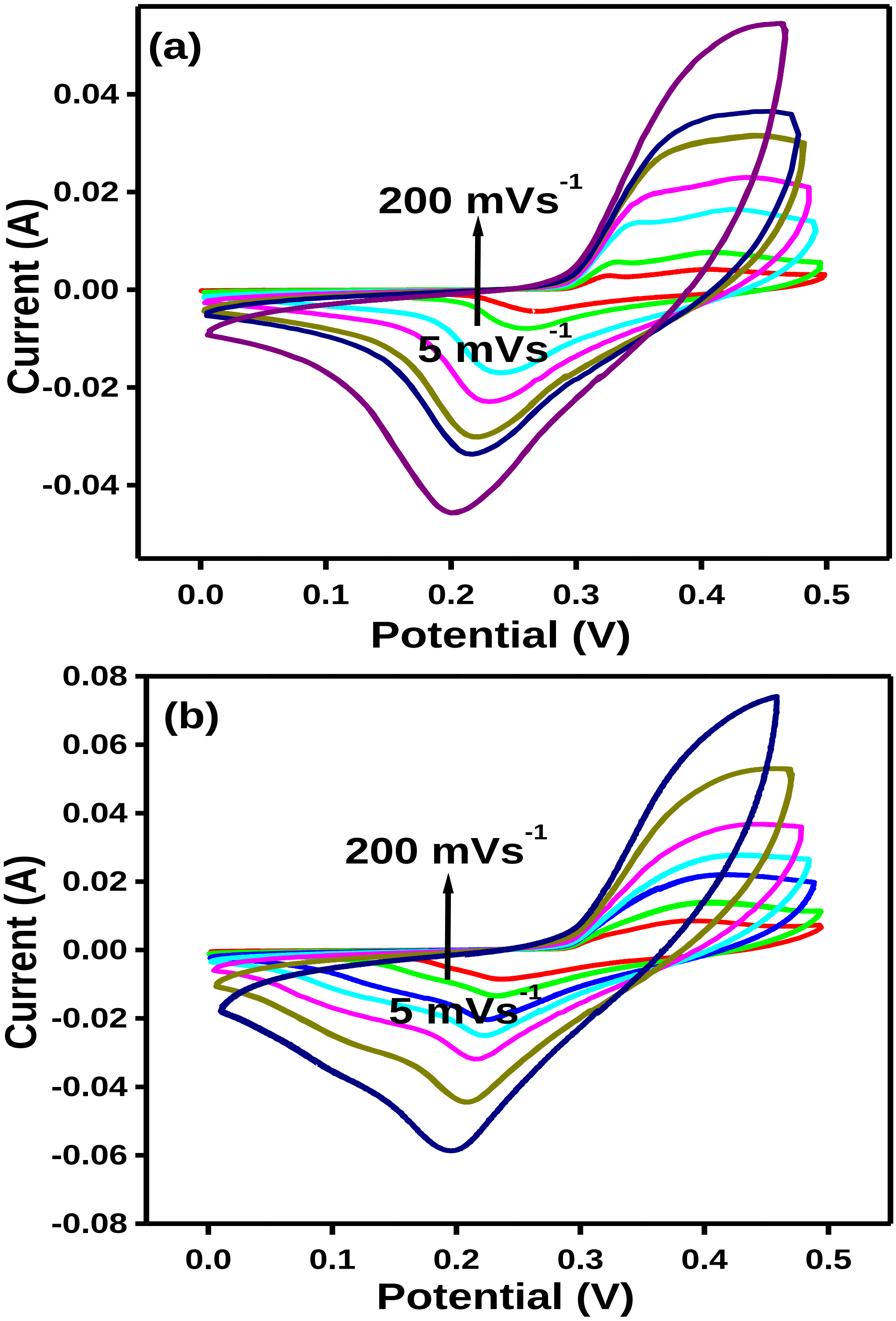

Figure S6. (**a**) Cyclic voltammograms of β-Ni(OH)$_2$-4 and (**b**) β-Ni(OH)$_2$-8 at a scan rate of 5-100 mV s^{-1}.

Galvanostatic Charge discharge curves of β-$Ni(OH)_2$-4 and (b) β-$Ni(OH)_2$-8.

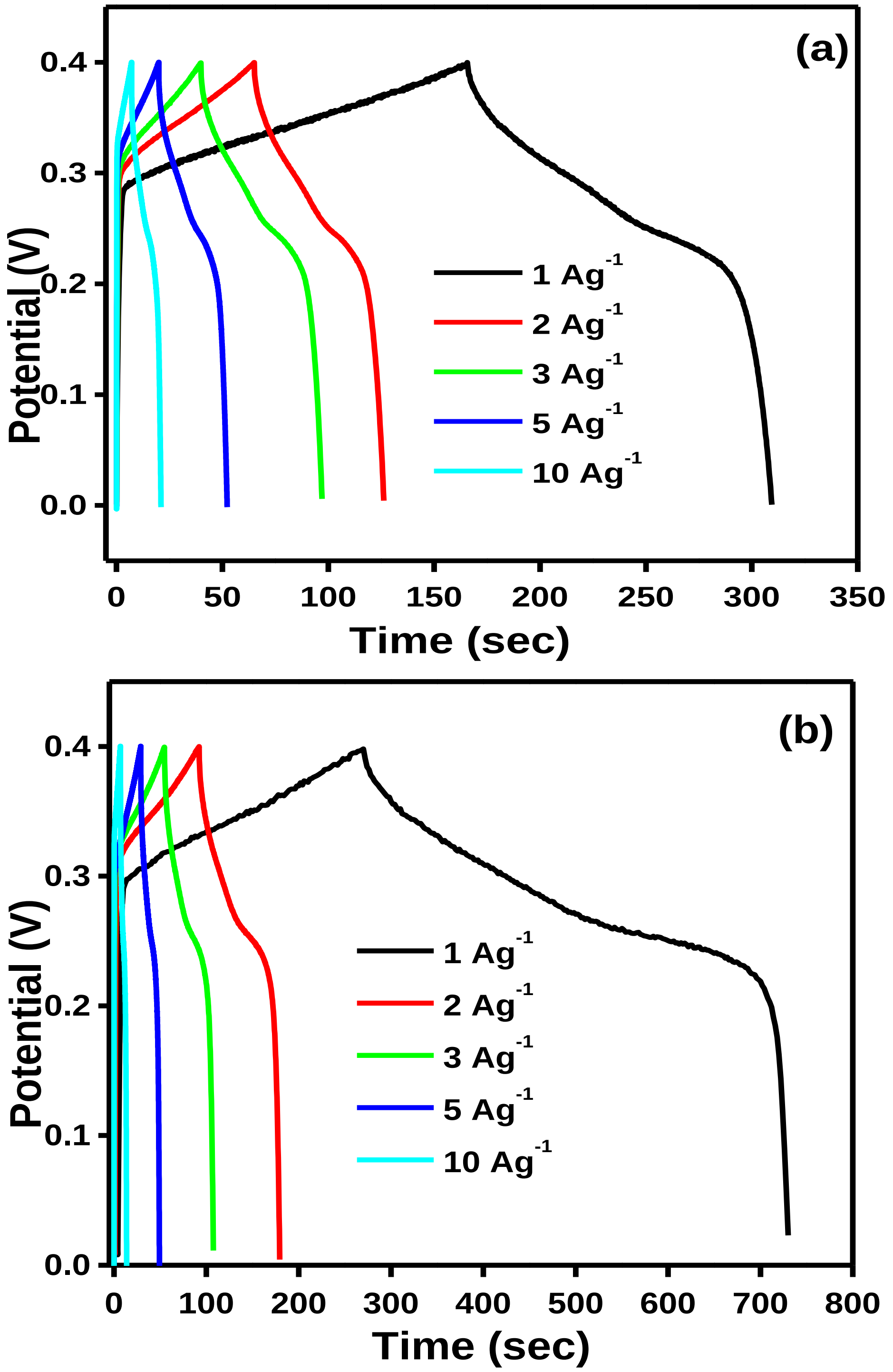

Figure S7. (**a**) Galvanostatic CD curves of β-$Ni(OH)_2$-4 and (**b**) β-$Ni(OH)_2$-8 at different current density.

Schematic illustration of 3D flower like β-$Ni(OH)_2$ used in charge discharge process.

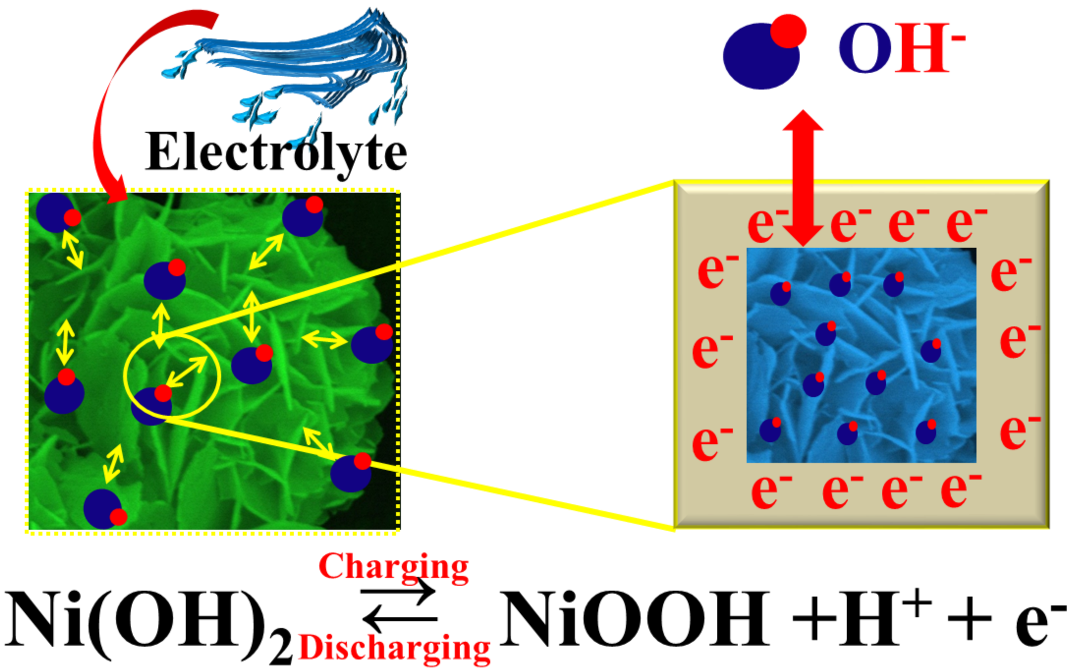

Figure S8. Schematic illustration of 3D flower-like β-$Ni(OH)_2$ used in charge discharge process.
